# A rare case of bilateral sequential spermatocytic seminoma

**DOI:** 10.1186/1477-7819-11-175

**Published:** 2013-08-06

**Authors:** Ning Xu, Fubiao Li, Runhui Tian, Mingming Shao, Lingyun Liu, Kaimin Guo

**Affiliations:** 1 Department of Urology, First Hospital of Jilin University, Changchun, Jilin, China; 2 Department of Andrology, First Hospital of Jilin University, Changchun, Jilin, China; 3 Department of Psychology, First Hospital of Jilin University,, Changchun, Jilin, China

**Keywords:** Spermatocytic seminoma, Fine-needle aspiration cytology, Bilateral spermatocytic seminoma, Testicular cancer

## Abstract

Spermatocytic seminoma (SS) is a rare testicular neoplasm characterized by a palpable, painless, slowly enlarging mass in the testis. Even more rare is a synchronous bilateral presentation. Only eight cases of bilateral SS have been reported in the literature, of which three cases were present with synchronous testis enlargement, and five were sequential. Here, we report an additional case of synchronous bilateral SS and present a comprehensive relevant literature review concerning clinical features, histopathology, and treatment.

## Background

Spermatocytic seminoma (SS), a rare testicular neoplasm differing from classical seminoma, usually presents in older men (>50 years of age) and occurs only sporadically in younger men (<30 years of age) [1-3]. SS has no equivalent ovarian carcinoma, is not associated with other types of germ cell neoplasia, and is not found in cases of cryptorchidism [4]. Depending on the different series, it represents 2 to 12% of all seminomas [5]. Since the first case of SS was described by Masson more than 50 years ago [6], more than 200 cases have been reported, most of them benign [7]. It is a solid tumor found solely in the testis with long duration of symptoms, presentation evident at an early stage, absence of metastasis, and bears an excellent prognosis [8]. Moreover, SS arises more commonly in the right testis, and has a higher frequency of bilateral occurrence than classical seminoma [9]. Following a thorough review of the literature, we have found only eight reported cases of bilateral SS. Herein, we report an additional case of bilateral SS and present a relevant review of the literature concerning clinical features, histopathology, and treatment.

## Case presentation

**Figure 1:**
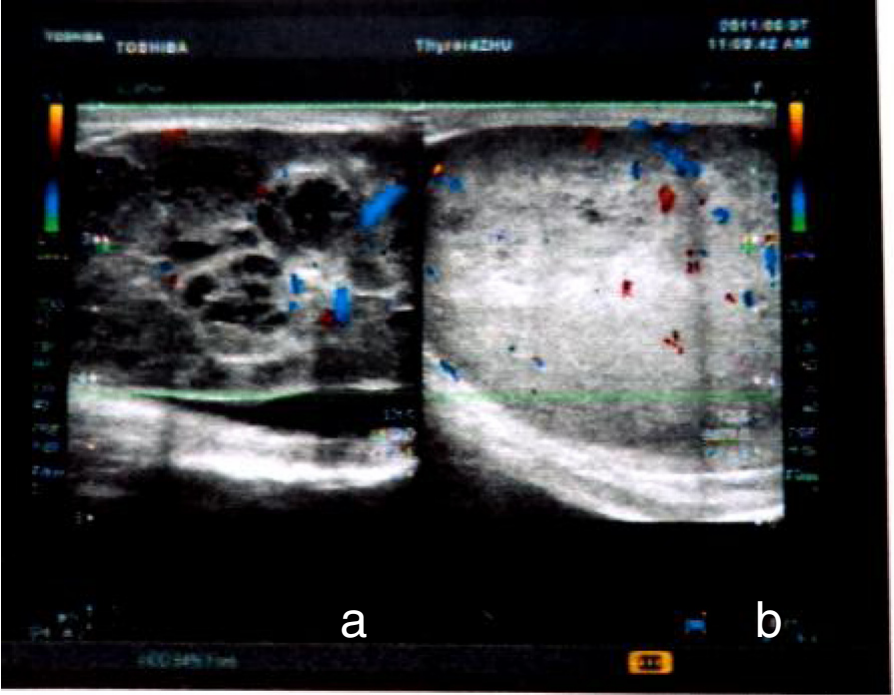
**Scrotal ultrasonography of bilateral SS. (a)** A left testicular multilocular tumor, **(b)** a right testicular solid tumor.

A 48-year-old man presented in June 2011 complaining of gradually increasing left testicular painless swelling for 2 years. For the immediate 2 previous months, he found his right testicle enlarging rapidly. There was no history of cryptorchidism, bilateral scrotal pain, voiding complaints, local trauma, weight loss, or hereditary disease. A comprehensive physical examination revealed bilateral testis enlargement and displayed firm consistencies to palpation. A huge, well-defined, non-tender, left testicular mass was palpable in the scrotum. The patient’s right testicle had similar palpatory findings as his left testicle, while his inguinal lymph nodes were not palpable. Scrotal ultrasonography (8 to 12 linear array transducer, TOSHIBA NemioXG, Tokyo, Japan) revealed a well-defined 75 × 45 × 40 mm left testicular multilocular tumor and an 80 × 50 × 45 mm right testicular solid tumor with heterogeneous echogenicity (Figure 1). Computed tomography of the abdomen and pelvis was negative for lymphadenopathy or other metastases. Clinically, the preoperative diagnosis was bilateral testicular tumor, with the possibility of classical seminoma.

**Figure 2:**
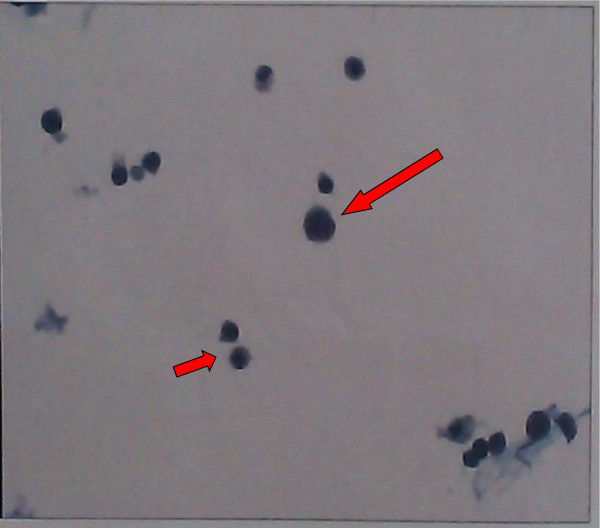
**Scrotal ultrasound fine-needle aspiration cytology revealed giant cells with large nuclei and coarse chromatin (long arrow).** Some intermediate-sized cells were noted (short arrow).

Taking into account the age of the patient, fine-needle aspiration cytology (FNAC) was performed. The cytological smear revealed malignant tumor cells present in both testes with bloody fluid background (Figure 2). The tumor markers alpha-fetoprotein, human chorionic gonadotropin, and serum lactate dehydrogenase were within normal limits. The patient then underwent left sided orchiectomy via inguinal approach, and the intraoperative frozen-section biopsy revealed SS. Subsequently, a right radical orchiectomy to remove the testicle was performed.

**Figure 3:**
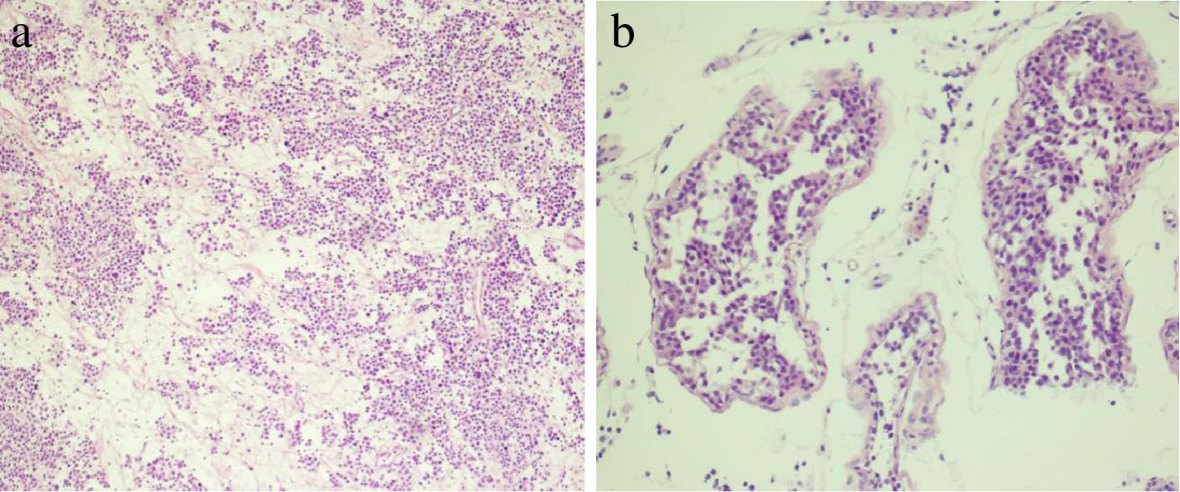
**Low-power photomicrograph of SS showing minimal to no lymphocytic infiltration. (a)** Less adhesion and interstitium among cells as well as extensive edema are observed by H&E staining (×40). **(b)** Spermatocytic seminoma infiltrating the seminiferous tubules (×100).

**Figure 4:**
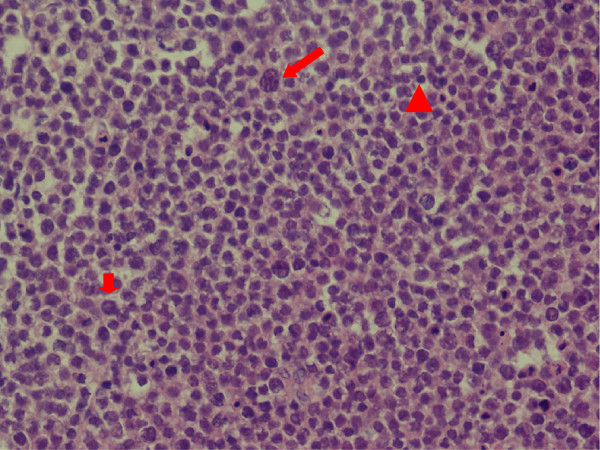
**High-power photomicrograph of bilateral SS.** Three types of cells were noted: large cells having characteristic ‘spermine’ chromatin with filamentous nuclear structure (long arrow); intermediate-sized cells which exhibit a finely granular chromatin with eosinophilic cytoplasm (short arrow) and small lymphocyte-like cells with scanty cytoplasm and a round nucleus (arrow head) (×40).

**Figure 5:**
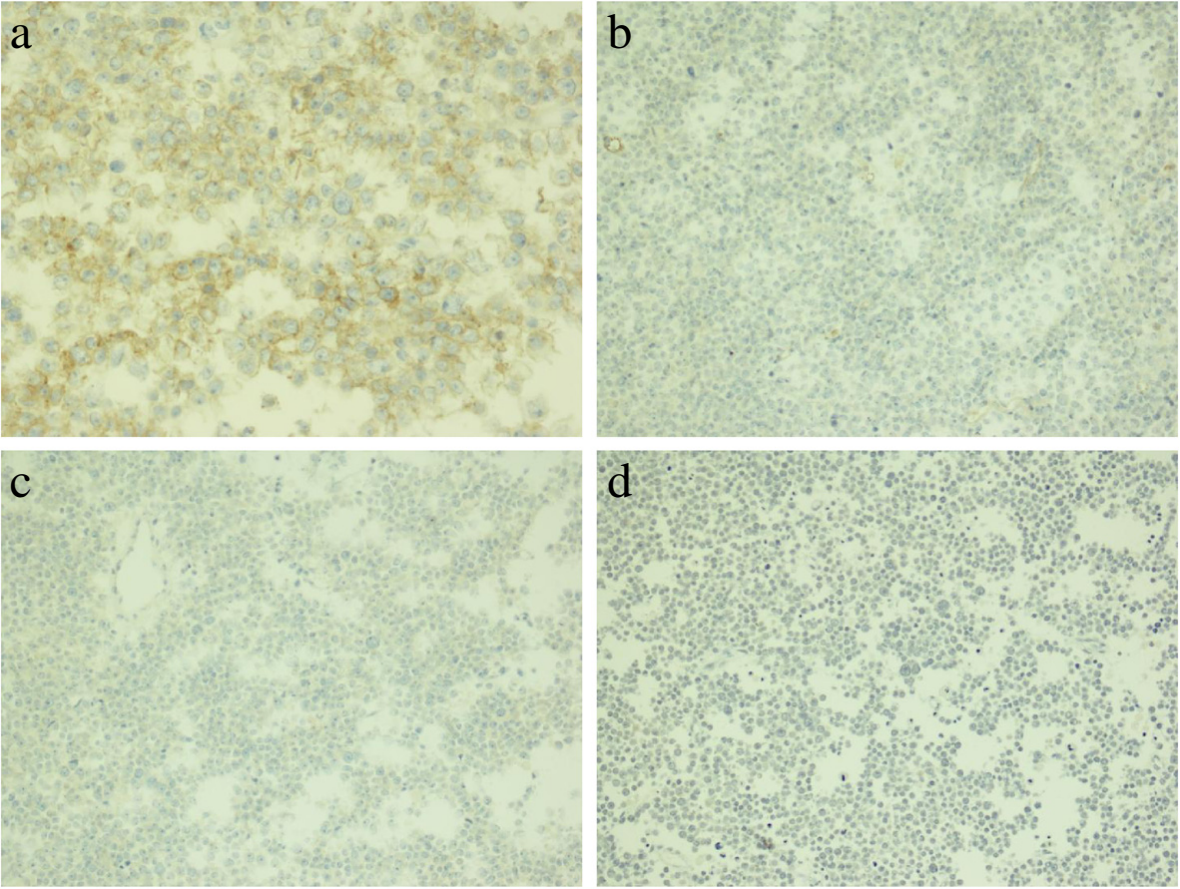
**Immunostaining of SS. (a)** Spermatocytic seminoma cells show cytoplasmic positivity with CD117 (×400), **(b)** negativity with OCT3/4 (×200x), **(c)** negativity with placental-like alkaline phosphatase (×200), and **(d)** negativity with CD30 (×100).

On gross examination, the left testicle measured 7 × 4 × 3 cm and weighed 108 g, while the right testicle measured 7 × 4 × 4 cm and weighed 118 g. Both masses had fleshy, pale-grey cut surfaces and were confined to the testis with no invasion or penetration of the tunica. There were no overlying skin changes. A histological examination confirmed the SS (Figures 3 and 4) and all surgical margins were not infiltrated. Immunohistochemical analysis revealed the tumor cells were positive for CD117 and Ki-67 (50% stain positively), and negative for CD3, CD20, CD30, placental-like alkaline phosphatase (PLAP), OCT3/4, S-100, and HMB45 (Figure 5). The data collected from immunohistochemical and histological analyses were consistent with the final pathologic diagnosis of bilateral SS.

Following the operation, the patient was followed closely without any adjuvant therapy and was in good condition with no evidence of metastasis 18 months after the operation.

## Discussion

SS is a distinctive, uncommon testicular germ cell neoplasm, first described by Masson in 1946 [6]. An extensive literature review revealed that the incidence of SS varies between 1.1 and 12% of all seminomas [4,10] and 1% of all testicular neoplasms [11]. The incidence of bilaterality, either synchronous or metachronous, is nearly 10% of cases [12]. To date, eight cases of bilateral SS have been reported, including one from India [13], four from Japan [14], and three from the USA [10,15,16]. We present the first case review of bilateral SS, including patient age, symptom duration, tumor size, treatment, and outcomes (Table 1).

**Table 1:** Characteristics and clinical course of published cases of bilateral SS

**Case No.**	**Age (years)**	**Duration of symptoms (month)**	**Size or tumor long axis**		**Treatment**	**Follow-up (months)**	**Outcome**
1^10^	68	NA	L.s	45 mm	Surveillance	121	NRD
		NA	R.s	53 mm			
2^13^	50	60	L.s	130 × 80 × 60 mm	RO	10	NRD
		18	R.s	50 × 40 × 30 mm	RO		
3^14^	70	4	L.s	50 × 35 × 25 mm, 52 g	RO + C	12	NRD
		1	R.s	52 × 45 × 30 mm, 57 g	RO + C		
4^14^	49	1	L.s	50 × 40 × 40 mm, 64 g	RO + L	48	NRD
		2	R.s	60 × 45 × 27 mm, 67 g	RO		
5^14^	50	60	L.s	80 × 60 × 33 mm, 210 g	RO + R	44	NRD
		60	R.s	100 × 70 × 35 mm, 390 g	RO + R		
6^14^	56	15	L.s	36 × 33 × 30 mm, 65 g	RO	6	NRD
		15	R.s	24 × 23 × 25 mm, 53 g	RO		
7^15^	77	108*	L.s	116 mm	RO + R	60	NRD
		108*	R.s	54 mm	RO + R		
8^16^	28	12	L.s	NA	RO	12	NRD
		36	R.s	NA	RO		
Our case	48	24	L.s	80 × 50 × 45 mm, 108 g	RO	18	NRD
		2	R.s	75 × 45 × 40 mm, 118 g	RO		

* Associated with hydrocele. *L* lymph node dissection, *L.s* left side, *NA* not available, *NRD* no recurrence of disease, *R* radiotherapy, *RO* radical orchiectomy, *R.s* right side.

SS is found exclusively in the testis and is not associated with any known risk factors for germ cell tumors including cryptorchidism, subfertility, or gonadal dysgenesis [10]. These tumors originate from a post-natal germ cell [12]. The detection of proteins SCP1 and XPA, which are normally expressed in the primary and pachytene spermatocyte stages, provide a clue that the origin of SS is in a more differentiated cell than in classical seminoma [17]. The gain of chromosome 9 appears to be a consistent finding in all cases of SS, which is not found in classic seminomas; in addition, other genetic abnormalities include gain of X chromosome. A recent study confirmed that *DMRT1*, a male-specific transcriptional regulator, was a likely candidate gene for involvement in the development of SS [18]. Most cases of SS present as a palpable, painless, slowly enlarging mass in the testis; only one patient was referred because of massive hemorrhage to hydrocele and needed emergency management [19]. SS usually occurs in older white men, in their sixth decade of life. Specifically, Chung and colleagues reported the median age of patients with SS was 62 years (range 32 to 77 years) [10]. Raiss and colleagues found the median age to be 45 years [11] and Carrière and colleagues found SS occurred just as frequently in young men as in older men (median age 54 years) [5]. Our review indicated the median age of bilateral SS was 55.1 years (range 28 to 77 years), with only one patient presenting at the much younger age of 28 years of age [16]. Therefore, while rare, the diagnosis should be considered even in young patients. Of the eight reported cases, three were present with synchronous testis enlargement and five were present sequentially. The average duration of onset was 35.5 months on the left testicle (range 1 to 108 months) and 30.3 months on the right testicle (range 1 to 108 months). Macroscopically, SS presents grossly as a testicular mass with a size ranging from 1.5 to 28 cm [20] and is generally a homogenous, well-circumscribed, solid, pale-grey or pink-tan tumor that is always confined to the testis. However, the rhabdomyosarcomatous transformation was seen infiltrating and destroying the rete testis and epididymis [21]. Matoska and Talerman reported that six SS patients with sarcoma developed metastatic disease, and the metastases most frequently affected the lung, liver, and retroperitoneum [22]. In our review, all tumors were classical SS and confined to the testis. The tumor size ranged from 36 to 130 mm with an average of 73.4 mm on the left and from 25 to 100 mm with an average of 58.6 mm on the right.

Histopathologically, the presence of sheets and cords of a mixture of different sized cells characterize SS with similarities to spermatogonia and spermatocytes [23]. These often have the typical spireme-like lacy chromatin distribution. Intermediate and large cells may have filamentous nuclear chromatin. The nuclei of all the cell types of SS are generally round or slightly oval and with a smooth contour, in contrast with the nuclei of classical seminoma cells, which are generally described as irregularly shaped and “squared-off” [20]. There are many case reviews or case reports that have described unilateral SS associated with rhabdomyosarcomatous or sarcomatous differentiation and anaplastic variant [6,7,20-22,24]. However, bilateral sarcomatous transformation have never been reported. In this case report, three populations of cells and a preponderance of intermediate-sized cells were noted in a clean background, with small cells with a dense hyperchromatic lymphocyte-like nuclei also present.

In most cases, classical seminomas show diffuse positivity for CD117, PLAP, and OCT4. However, many of the markers useful in other types of germ cell tumor are generally negative in SS [25]. Positive c-Kit (also known as CD117) staining is controversial. While Raiss and colleagues showed four cases of SS were all negative for CD117 [11], Decaussin and colleagues found c-Kit was expressed in 100% of seven SS cases [26]. C-Kit positivity was also shown by Dundr and colleagues [6] and Narang and colleagues [21]. In total, c-Kit is positive in around 40% of all cases in the literature, a number consistent with our findings in this study. P53 overexpression may be involved in the pathogenesis anaplastic SS, due to evidence in two cases in a study [24]. However, this hypothesis needs molecular pathology confirmation. The Ki-67 index, the proliferative activity with monoclonal antibody MIB-1, may show nuclear positivity in about 10% of tumor cells and 30 to 40% of tumor cells in areas with anaplastic features [6]. In our case, the Ki-67 index was 50%. The specific sperm cell lineage marker VASA can be positive in both classical and SS, but the staining is usually more intense in the latter [27].

A preoperational SS diagnosis is unlikely due to its rarity and similarity with classical seminoma. Saran and colleagues applied FNAC to diagnose a case of SS and confirmed it by histology [28]. The authors identified three types of cells, with a preponderance of medium-sized cells present in a clear background; low mitotic rate and absence of lymphocytes were also noted in aspirational smears. Lopez and Aranda also concluded FNAC was a simple diagnostic procedure for providing the confirmative diagnosis of SS [29]. In our patient, preoperative diagnosis was also a challenge as the aspirational fluids were so scarce that cytological testing was obscure with only a few malignant cells found.

Radical orchiectomy via an inguinal approach followed by adjuvant is a typical malignant testicular tumor therapy treatment. Although sporadic cases of metastatic SS have been reported, SS is an indolent neoplasm that rarely metastasizes and bears an excellent prognosis [10]. Hence, orchiectomy alone is indicated for treatment. However, when sarcomatous dedifferentiation is involved, aggressive behavior, presence of metastasis or poor outcome may result. A handful of SS cases have been described that have transformed to rhabdomyosarcoma [7,21,22]. In such settings, adjuvant chemotherapy and radiotherapy may be beneficial. In bilateral SS cases, one case underwent chemotherapy and two received radiotherapy after orchiectomy [14,15]. There was only one patient who received no intervention except surveillance. With a median follow-up of 36.8 months (range 6 to 121 months), no patient has relapsed. Post-orchiectomy surveillance comprising 6-monthly chest radiographs and tumor maker assays for the first 3 years in our practice should be advocated.

## Conclusions

SS is a rare testicular tumor, with bilateral sequential SS presentation being even more rare. It differs from classical seminoma especially by its behavior, characterized by an almost complete inability to metastasize with only very few examples described with metastatic behavior. FNAC is a procedure used for precluding malignant tumors. However, histopathology is essential to confirm the diagnosis. In spite of scarce evidence of bilateral SS metastasis, long-term periodic surveillance remains necessary.

## Consent

Written informed consent was obtained from the patient for publication of this case report and any accompanying images. A copy of the written consent is available for review by the Editor-in-Chief of this journal.

## Abbreviations

FNAC: Fine-needle aspiration cytology; H&E: Hematoxylin and eosin; PLAP: Placental-like alkaline phosphatase; SS: Spermatocytic seminoma.

## Competing interests

The authors declare that they have no competing interests.

## Authors’ contributions

NX and FL performed the operation; RT and MS collected relevant literature and modified the draft; KG wrote the initial draft; LL checked the manuscript; all authors read and approved the final manuscript.
